# Active learning in the lecture theatre using 3D printed objects

**DOI:** 10.12688/f1000research.7632.2

**Published:** 2016-06-03

**Authors:** David P. Smith

**Affiliations:** 1 School of Bioscience and Chemistry, Sheffield Hallam University, Sheffield, UK

**Keywords:** 3D printing, Active Learning, Experiental Learning, Higher Education, Biomolecules

## Abstract

The ability to conceptualize 3D shapes is central to understanding biological processes. The concept that the structure of a biological molecule leads to function is a core principle of the biochemical field. Visualisation of biological molecules often involves vocal explanations or the use of two dimensional slides and video presentations. A deeper understanding of these molecules can however be obtained by the handling of objects. 3D printed biological molecules can be used as active learning tools to stimulate engagement in large group lectures. These models can be used to build upon initial core knowledge which can be delivered in either a flipped form or a more didactic manner. Within the teaching session the students are able to learn by handling, rotating and viewing the objects to gain an appreciation, for example, of an enzyme’s active site or the difference between the major and minor groove of DNA. Models and other artefacts can be handled in small groups within a lecture theatre and act as a focal point to generate conversation. Through the approach presented here core knowledge is first established and then supplemented with high level problem solving through a "Think-Pair-Share" cooperative learning strategy. The teaching delivery was adjusted based around experiential learning activities by moving the object from mental cognition and into the physical environment. This approach led to students being able to better visualise biological molecules and a positive engagement in the lecture. The use of objects in teaching allows the lecturer to create interactive sessions that both challenge and enable the student.

## Introduction

Ability to conceptualize 3D shapes is central to the understanding of biological processes. The dogma that the structure of biological molecules leads to function is central to biochemical understanding and is a core principle of the field. For example how the binding site of enzymes catalyses a reaction or how the major groove of DNA allows specific interactions with transcription factors. Understanding of such concepts is often a requirement for accreditation by learned bodies such as the Society of Biology, 2013 and the Institute of Biomedical Science, 2010 ( https://www.ibms.org/go/qualifications/ibms-courses/accreditation). Grounding in these concepts is undertaken during the first year of study on undergraduate courses within core modules in large group teaching environments. Students arrive with a range of experiences and prior knowledge from the basic to a more in-depth understanding of these molecules. The use of physical models as a learning tool has been used to depict molecular geometry in both biochemistry and chemistry ( Dori & Barak, 2001). The benefit of these objects has been demonstrated in an analysis of visuospatial thinking in chemistry. In this study it was concluded that adept visual perception skills correlate with achievement ( Dori & Barak, 2001). DNA and protein models assembled from coloured computer-printouts on transparency film sheets have also been demonstrated to aid students in grasping various aspects of biopolymers ( Jittivadhna *et al.*, 2010). Students who hold physical models in their hands gain a better understanding of molecular geometry than they could achieve solely from viewing images on a printed page. It has been reported by both Herman *et al.* (2006) and Bain *et al.* (2006) that physical models that can be easily manipulated can play an important role in capturing the interest of students. These models encourage deeper sophisticated thinking ( Bain *et al.*, 2006). Additionally, the students gain a language for talking about the concepts in question and can enhance their understanding of abstract concepts by the handling of models ( Wu & Shah, 2004).

The use of computer based representations is an alternative way to visualise biological molecules. There are a wide range of 3D visualisation programs available which enable molecular structures to be moved, altered, rotated and interrogated. Programs such as the Java based Jmol applet allow students to manipulate molecules and investigate their structures. There are also a range of free programs that allow students to visualise molecules on smart phones and tablets such as "Molecules" for Apple iOS and "ESmol" for Android devices. These programs offer many advantages such as ready access to the > 118500 structures available in the protein data bank (PDB). However, their use as tool in large group teaching can be problematic as all students require access to a device. In addition providing technical support to 150 students across three different platforms within a lecture theatre can be problematic. Molecular viewers do work very well in small group seminar or lab sessions where support can be given or devices can be provided. Students can be directed through a range of tasks and the molecules can be explored in greater depth ( Harris *et al.*, 2009). However, the tablet based applications lack the tactile aspect of physically handling the object in question and can prevent the abstract observations that generate the initial conversations. 3D printed models are also engaging and can be used by groups of people who have had little training, unlike visualisation programs ( Harris *et al.*, 2009). Handling objects facilitates the ability to quickly make abstract observations for example the spiral nature of DNA or the large clefts on an enzyme. In a study in which 3D printed models were offered alongside a molecular imaging program the students tend to prefer the models when asked questions about molecular structure that required higher order thinking skills ( Harris *et al.*, 2009). The tactile nature of the models appears therefore to lead to a more lasting memory of the interaction (Hurman, 2006).

Eysenck (2012) explains that within the teaching space students are required to imagine what would happen if an object such as a molecular model was rotated or altered in a process known as "mental rotation". Although some students have the ability to picture 3D objects in their minds, this is not true for all. The handling of molecular models within teaching sessions can aid this mental rotation and the use of models falls within the theories of object based learning. This approach involves the active integration of objects into the learning environment ( Chatterjee & Hannan, 2015). The idea that working with objects strengthens learning is the central proposition of object-based learning, according to Romanek & Lynch (2008). It has been suggested that the sense of touch can lead to a more memorable learning experience. The use of museum artefacts in history, art and biology has been well explored and there are a number of parallels between the handling of objects within these disciplines. Object-based learning theory links student activity to meaning by challenging the student to engage with and interrogate the object. It represents a constructivist approach in which the students develop their knowledge and understanding though interaction ( Chatterjee & Hannan, 2015). While the teacher facilitates this learning, the students have to learn for themselves through their interaction with each other centred on the object ( Hannan *et al.*, 2013). This approach enables the student to explore processes and events related to the object and further link these observations to complex abstract ideas and concepts.

Traditionally the knowledge required to understand 3D structure and related concepts within biochemistry have been presented through the use of PowerPoint slides that maybe heavy in text. Slides represent objects two dimensionally and this is can be useful for detailing core knowledge. This approach however does not help the students develop more complex cognitive 3D mental rotation skills ( Nigel, 2014). There is a danger that two dimensional visualisations engage students more superficially. Content can be perceived as supporting a didactic and passive transfer of information and fails to support the development of knowledge and deep understanding ( Biggs, 1999). This behaviourist approach has its merits under certain conditions, such as when a large amount of content needs to be covered in a short amount of time ( Woolfolk, 2009). However, this approach is restricted to the acquisition of knowledge and can prevent access to higher tiers of learning ( Anderson *et al.*, 2000; Bloom, 1956). An alternative to this approach is the inclusion of active learning in teaching sessions whereby students become involved in the learning and are engaged in activities leading to higher order thinking (analysis, synthesis, evaluation) ( Bonwell & Eison, 1991). One such active approach is presented here in which students handle physical 3D printed objects within a large group teaching setting. The students report that this approach allows them to visualise the molecules in question and aids in their understanding of function.

## Methodology

Ethics for this study was acquired through self-regulation following the Sheffield Hallam University Research Ethics Policy. Given the nature of the work further ethical approval was not seen as necessary after the initial scrutiny as no identifiable, confidential or controversial information would be collected.

Within the research setting physical structural models of molecules have long-been used to help understand function. Models of the protein in question are often generated and handled in small group meetings as talking points to generate new hypotheses. This approach was adapted to large group teaching sessions with cohorts of 150 students in their first year of study delivered in a standard tiered lecture theatre. Sessions using the models were delivered twice to the same students on their first year of study and have been run twice a year for three consecutive years. Delivery occurred once in the first semester and once in the second semester of a core biochemistry module. The models have also been used in second year teaching when discussing DNA binding proteins within a Molecular Biology module. The students were presented with the main concept that the structure of a molecule brings about its function. Existing sessions were adapted to deliver core knowledge supplemented with high level problem solving through the use of 3D printed models to encourage student engagement in learning. The 3D printed molecules in question were linked to the core content and act as a focal point for learning.

**Figure 1.**
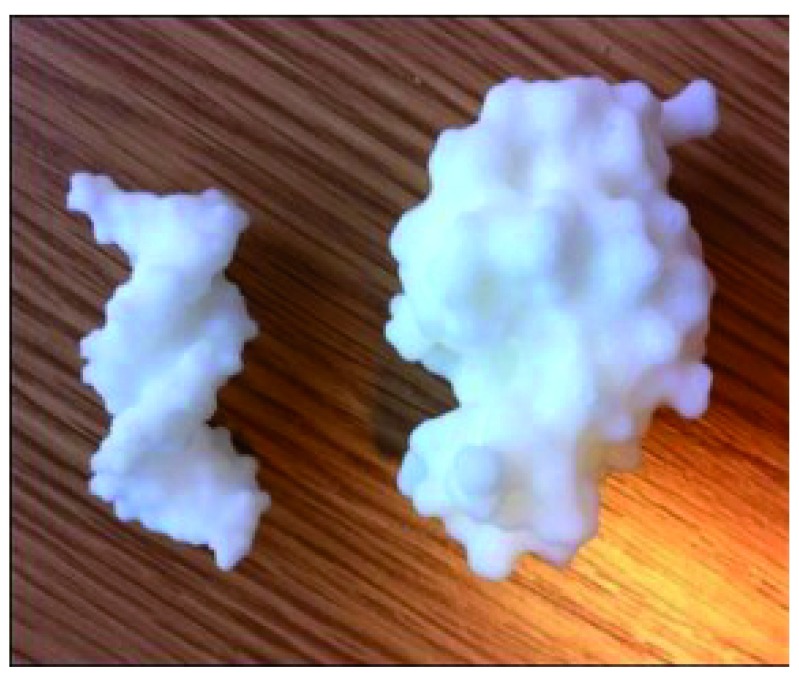
3D printed models. B-form DNA (left) and the enzyme lysozyme PDB: 2LYZ (right) used within the teaching session.

**Figure 2.**
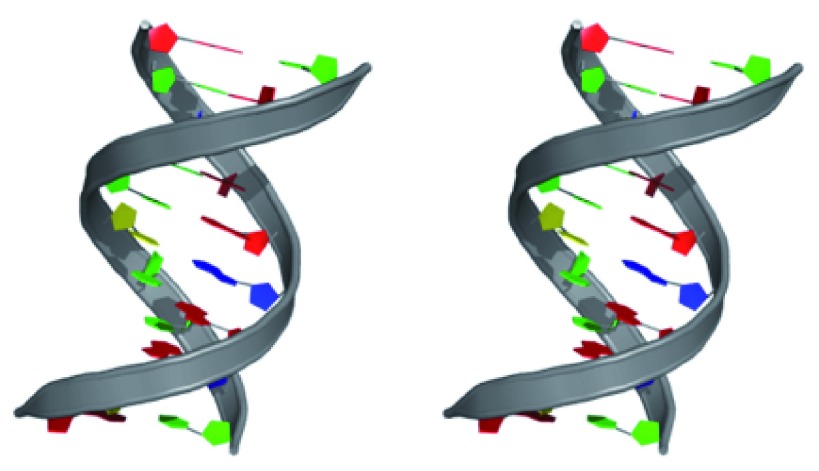
Handout example. Cross eye stereo image: The instruction to students was to gaze at the stereo pair, keeping your eyes level (don’t tilt your head left or right), and cross your eyes slightly so that the two images in the centre come together. When they converge or fuse, you will see them as a single 3D image.

Models were created from the protein data bank (PDB) code 2LYZ (Lysozyme) and B-form of DNA taken from the now defunct Glactone Pedagogical PDB collection. The PDB file was modified by the removal of the water molecules and the surface of the molecule was calculated in a molecular graphics program (Visual Molecular Dynamics 1.8.5). PDB files are also included as Supplementary material 1 and Supplementary material 2. The resulting files were rendered in a standard STL format using the (STL Plugin, Version 2.0) which is compatible with CAD and most 3D printers. STL files are also included as Supplementary material 3 and Supplementary material 4. Models used here were produced on a fused deposition modelling (FDM) Dimension sst 768 rapid prototyping 3D printer ( Figure 1) and were approximately 2 × 2 × 4 cm. Twenty models of each type were made and cost ~£7 each for the raw materials. The DNA models were scaled such that an index finger would fit into the major grove. An understanding of how other molecules, such as transcription factors, interact with DNA and bring about changes in transcription and translation are key learning points in biochemistry and are central to molecular biology. Lysozyme was selected because its structure and function are well understood being the first enzyme for which a structure was determined ( Blake *et al.*, 1965). It also has a clearly defined active site in which the substrate has been modelled. Paper-based stereo images were also provided in the same session to be taken away and viewed later. The use of the 3D projection images also allowed the students to review and reflect on the learning at a later date and gave a focal point and prompt for later revision ( Figure 2).

In the DNA sessions the main learning outcome was to understand the difference between the major and minor grove within the structure of DNA and how proteins interact with these conformations. Within the Lysozyme sessions the learning outcome was to be able to identify where an active site maybe located on an enzyme and how a substrate would interact with it. Sessions were structured so that taught content prepared the students for the learning activities by first establishing core knowledge. This content gave the students the vocabulary they needed to later describe the objects they would handle. The taught content laid the foundation knowledge relating to how molecules such as enzymes perform reactions and an appreciation of the structure of DNA. The active learning component was then included within the sessions to place the object into a physical, rather than cognitive space. This was achieved by allowing the students to handle the objects and physically rotate and view the 3D printed models of these biomolecules.

### Using Kolb's Experiential Learning Cycle to understand the approach

The overall teaching style follows a simplified form of Kolb’s experiential learning cycle ( Kolb, 1984). This model is well-established in science based learning. As teachers and learners we are able to jump onto the cycle at any point but in order for it to be useful the stages must be followed in sequence. Learning can then be applied in new situations and subsequently built upon. The approach included thinking, doing, feeling and reflecting stages.

### Thinking (abstract conceptualization)

New concepts were introduced through the use of slides, videos and written material. A range of media animations, web-based content and strong links to core texts were used. The "thinking" section of the lesson plan had prepared the students to identify key features of the models they would later handle.

### Doing (active experimentation)

**Figure 3.**
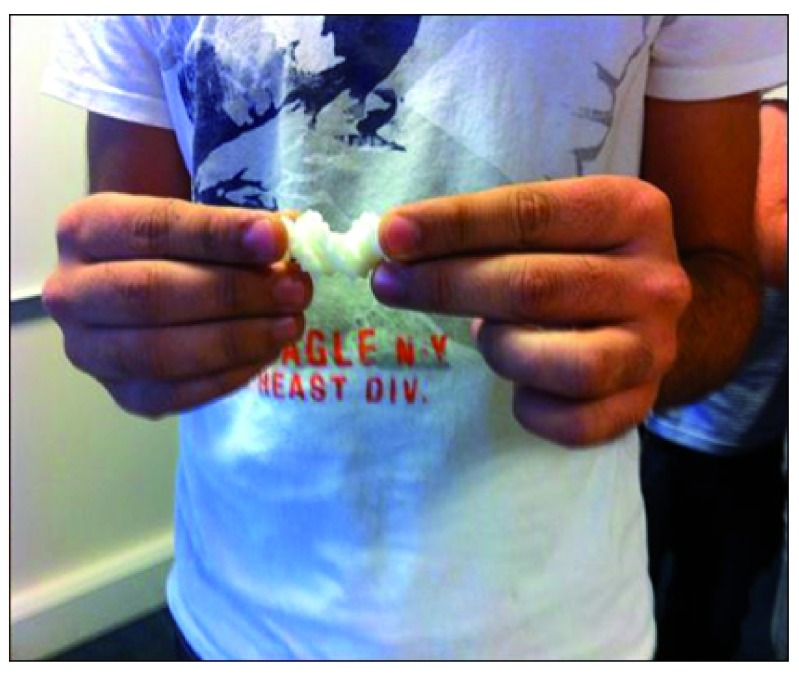
Photograph. Students handle the 3D printed molecules and were asked to identify structural features.

In order to develop a 3D understanding of biological molecules students were asked to handle printed models and apply their new knowledge and concepts through self-directed small group discussions ( Figure 3). Twenty molecular models were handed around the group of 150 students in a standard lecture theatre, with one lecturer running the session. They were handed out starting at the end of each of the rows of students. The students were asked to pass the molecules around and handle them directly. Whilst handling the objects the students were encouraged to talk with their peers about their observations. Questioning directed by the lecturer was centred on those features they could directly observe and was objective, such as: What do they feel like? What general shape do they have? What features can you observe? This encouraged student interaction as there was no wrong answers to the questions as it was personal observation.

### Feeling (concrete experience)

Through this approach core knowledge is first established which is then supplemented with high level problem solving through "Think-Pair-Share" cooperative learning strategies with the lecturer acting as a facilitator. Students are asked to think through questioning about an aspect of the object and discuss the answers with each other. Questions were asked that probed understanding, such as what are those bumps on the surface? What is the function of that groove? As such, learning is enhanced through the opportunities to elaborate on the ideas through conversation. It was observed that this approach led to increased student engagement in the lecture theatre as the students are willing to talk with each other and the lecturer as confidence in their understanding increased.

### Reflective observation

**Figure 4.**
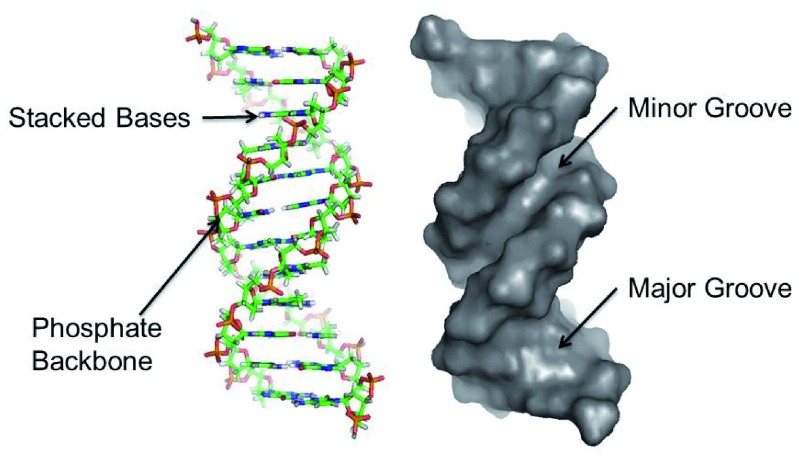
Handout example. Handouts were designed that allowed the students to identify key features of the molecule in question and complete a question sheet. This ensured key learning objectives were recorded.

Finally the students are given time and encouraged to write on handouts in their own words the key points and note theories that have been discussed ( Figure 4). The handouts were structured such that the key learning objectives were recorded (handouts used can be found in Supplementary material 5). For example, students were asked to identify key features of the molecule in question and complete a question sheet where they were asked to identify structural features. In order for the students to take ownership of the knowledge, they discussed specific situations for how this information is used in practice. Examples were given from a research-informed context and were tailored to be course specific.

## Results

Students in their final year of study were asked to reflect on the use of the 3D printed models during their past learning. 44 students were asked " *Did you find the models helpful, if so how?*" through an anonymous survey. Of the 44 responses 35 remembered using the models and 32 of these responded with positive comments (3 with neutral comments). Of the 9 students who did not remember using the models many admitted to not having attended the lecture.

Some students commented on the benefits the models provided as a visual aid,

" *they were very useful for highlighting the key lecture points as well as being a visual aid*."

Student comments also highlighted the use of the models as a tool in understanding the key learning objectives,

" *Very useful to help understand major and minor grooves*",

and

" *Allowed us to visualise the major and minor groove of DNA, as well as the binding sites for enzymes*".

The students also explained that the models provided an alternative way of presenting information,

*"they gave a better 3D understanding of the 3D structure of the enzyme than a 2D computer image.*"

It was also noted that the models could be used to gain a sense of scale, for example, the difference in size between a ribosome and organelles. The full list of comments is included as Supplementary Information 6. During each of the sessions there was a high level of student engagement involving the students talking to each other and to the lecturer with the key learning points being recalled three years after the teaching session.

## Conclusion

Access to 3D printing technology is becoming more wide spread as the costs associated with the technology drops. Active learning approaches are also becoming increasing common place as teaching staff move away from didactic strategies ( DesLauriers *et al.*, 2011; Seery, 2015; Sharples *et al.*, 2014).

The use of objects within the classroom is an example of an active learning approach and, in evaluations conducted with my own students, object-based learning was identify as being engaging and informative. Students describe how helpful the models were as visual aids such as “ *amazing and made proteins fun*” and “ *easier to visualise*” with reference to the 3D printed models. Such comments reinforce the concepts on which object based learning theory is based with the models stimulating conversation and aiding in the visualisation of the molecules.

The positive comments from students polled in this study, in which 3D printed models were rated as useful, are echoed in a comparable study where a side-by-side comparisons of seven different learning tools was undertaken by students in an introductory biochemistry class ( Roberts *et al.*, 2005). In that study, the models were shown to have an important role in capturing the interest of the students and stimulated sophisticated questioning. The main difference between these observations and those presented here, relate to the learning environment. Within the side-by-side comparisons study, the core concepts were introduced in a lecture and the models were handled as part of a 3-wk laboratory in a group of twenty students, generating observable learning gains. In the study discussed here, however, 150 students were allowed to handle the models directly within the lecture environment. Handling the models aided the students in visualising the molecules demonstrating the applicability of this approach to large group teaching. Schönborn & Anderson (2006) states that the ability to visualise ideas is a key skill for all students and it is a key skill for biochemists who are often presented with a range of visual interpretations including drawings, images, dynamic visuals, animated visuals, multimedia, and virtual reality environments. The use of models has the potential to help students construct their own visualisation and understanding of these molecules as demonstrated by the student comments reported here. The students engage with the models which stimulate conversation rather than distract attention.

The use of objects, can be seen as a focal point for conversation. This suggests there are similar applications to enhance other areas of teaching. Peers within the nursing team at my own university have considered the use of dolls as talking points for their students to support discussions about empathy. Such abstract learning environments dealing with relationships rather than facts and thinking situations in symbolic form can be pictured as an area of conceptual knowledge ( Anderson *et al.*, 2000). Objects have also been used by peers in analytical chemistry teaching demonstrating how parts of an instrument such as HPLC columns are used in drug detection.

Findings from my study support Chatterjee's (2015) conclusion that the students gain real knowledge by being actively involved in the experience of handling the objects. It has been highlighted that object-based learning should be both mentally and physically stimulating through some form of problem solving or experimentation ( Coffield *et al.*, 2004). This was achieved in this study by challenging the students to find the active site of Lysozyme or identify the region of DNA to which proteins bind.

While objects can be used to enhance learning, there are logistical and pedagogical barriers to implementing them. For example, Cain (2010) says the main barrier for the implementation in the biosciences within the lecture theatre is the challenge of adopting of a more student-centred and open-ended activities. The use of models in teaching also needs to expand the concepts as understood by students at a particular stage in their learning ( Bent, 1984). There is a danger for example when discussing enzymes that concepts such as the lock and key model could be reinforced over an induced fit model. Such dangers should be at the forefront of the lectures mind when utilising these models.

The use of artefacts in teaching opens new ways to engage and challenge students. Teachers can create interactive sessions that challenge students to see artefacts through the lenses of mathematics, science, language, arts, and social studies. While the use of objects in both large and small group teaching is currently under researched and under reported, it has the potential to increase student engagement by facilitating active learning methods.

## Supplementary material

Supplementary material 1: Lysozyme PDB file. The file "2LYZ no water.pdb" contains the atomic coordinates of the enzyme Lysozyme with the crystallographic water molecules removed from the file. The file originated was sourced from the PDB file 2LYZ.

.

Supplementary material 2: B-form DNA PDB file. The file "bdna.pdb" contains the atomic coordinates of B-form DNA used to create the DNA model structure and hand-out images. The file originated from the now defunct Glactone Pedagogical PDB collection.

.

Supplementary material 3: Lysozyme STL file. The file "lysozyme.stl" contain surface renderings of lysozyme and was used to create the 3D printed model shown in Figure 1. The files is in a standard STL (STereoLithography) format native to the stereolithography CAD software created by 3D Systems. This file format is supported by many software packages and is widely used for rapid prototyping and 3D printing.

.

Supplementary material 4: B-form DNA STL file. "B-DNA2.stl" contain surface renderings of B-form DNA and was used to create the 3D printed model shown in Figure 1. The files is in a standard STL (STereoLithography) format native to the stereolithography CAD software created by 3D Systems. This file format is supported by many software packages and is widely used for rapid prototyping and 3D printing.

.

Supplementary material 5: Hand-outs used to allow students to record their experiences and notes from the session.

.

Supplementary material 6: **Record of student responses.**
