# Supplementary material for: Active learning in the lecture theatre using 3D printed objects
Source: F1000Res. 2016 Jun 3;5:61. Originally published 2016 Jan 13. [Version 2] doi: 10.12688/f1000research.7632.2 (PMC4911624; doi:10.12688/f1000research.7632.2)
Supplement: Supplementary file 5 [file f1000research-5-9453-s0004.tgz › fc365ac6-621c-46bc-bef7-68638be3a877.pptx]

## Slide 1
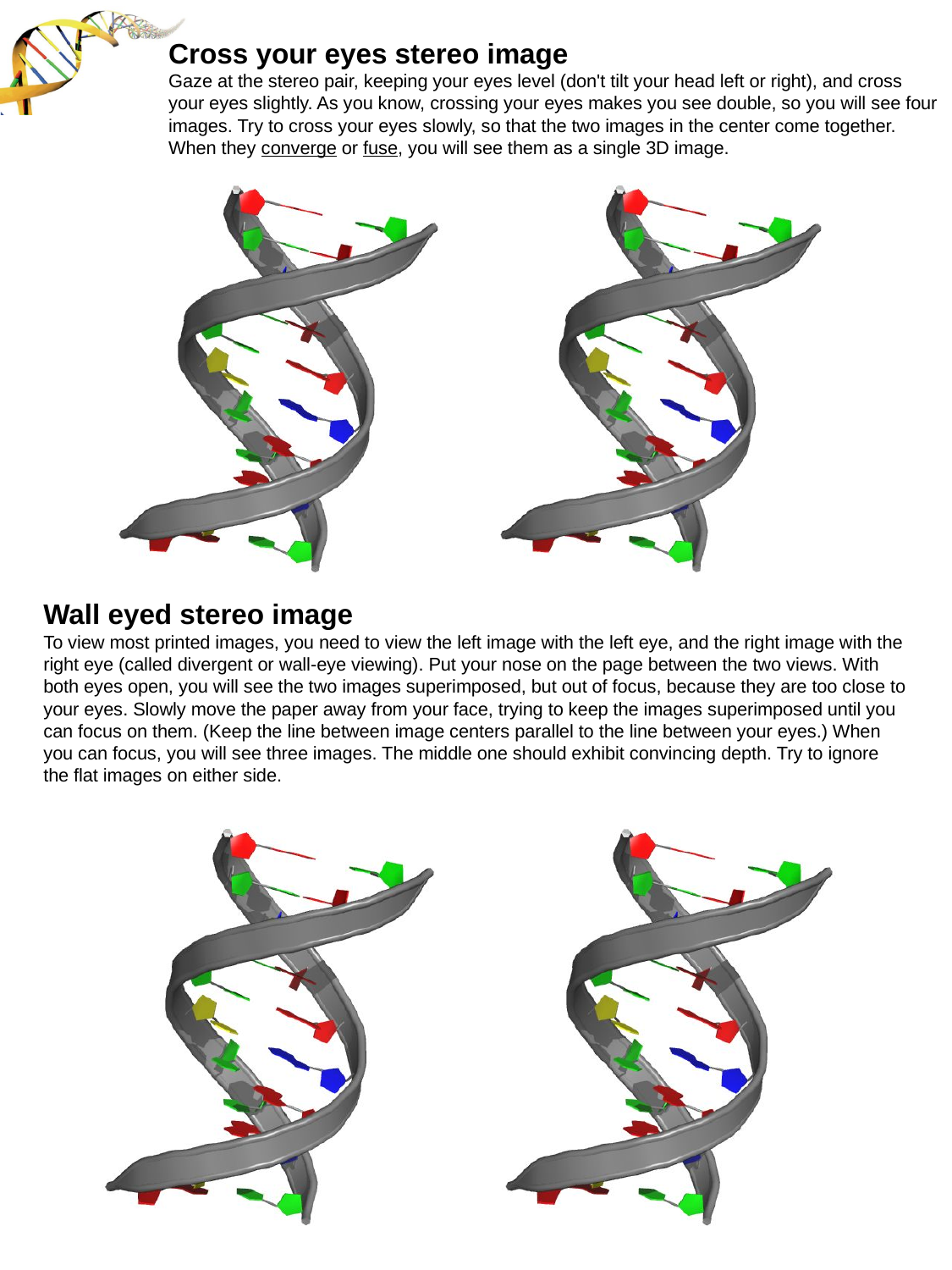

Cross your eyes stereo image
Gaze at the stereo pair, keeping your eyes level (don't tilt your head left or right), and cross your eyes slightly. As you know, crossing your eyes makes you see double, so you will see four images. Try to cross your eyes slowly, so that the two images in the center come together. When they converge or fuse, you will see them as a single 3D image.
Wall eyed stereo image
To view most printed images, you need to view the left image with the left eye, and the right image with the right eye (called divergent or wall-eye viewing). Put your nose on the page between the two views. With both eyes open, you will see the two images superimposed, but out of focus, because they are too close to your eyes. Slowly move the paper away from your face, trying to keep the images superimposed until you can focus on them. (Keep the line between image centers parallel to the line between your eyes.) When you can focus, you will see three images. The middle one should exhibit convincing depth. Try to ignore the flat images on either side.

## Slide 2
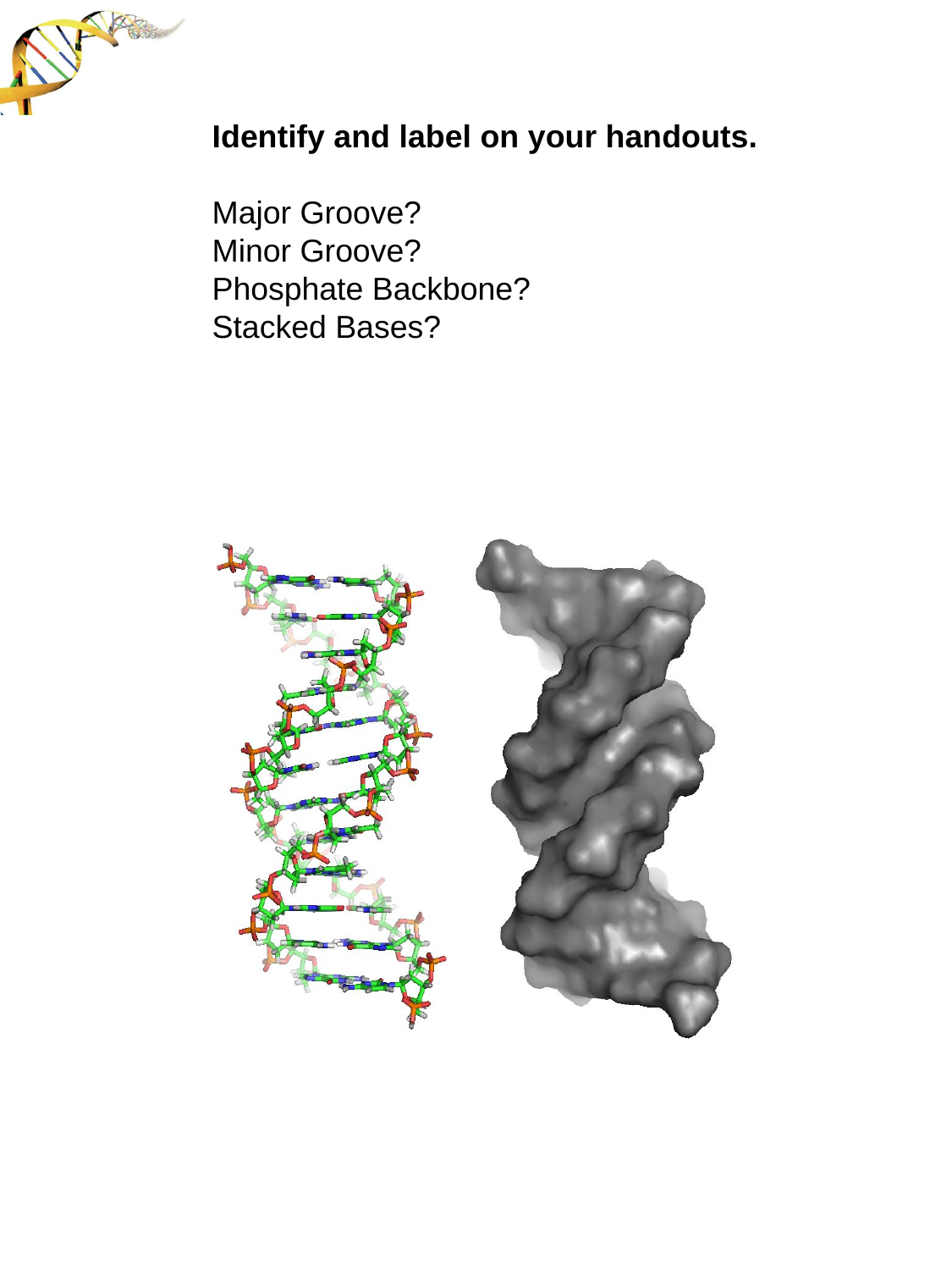

Identify and label on your handouts.
Major Groove?
Minor Groove?
Phosphate Backbone?
Stacked Bases?

## Slide 3
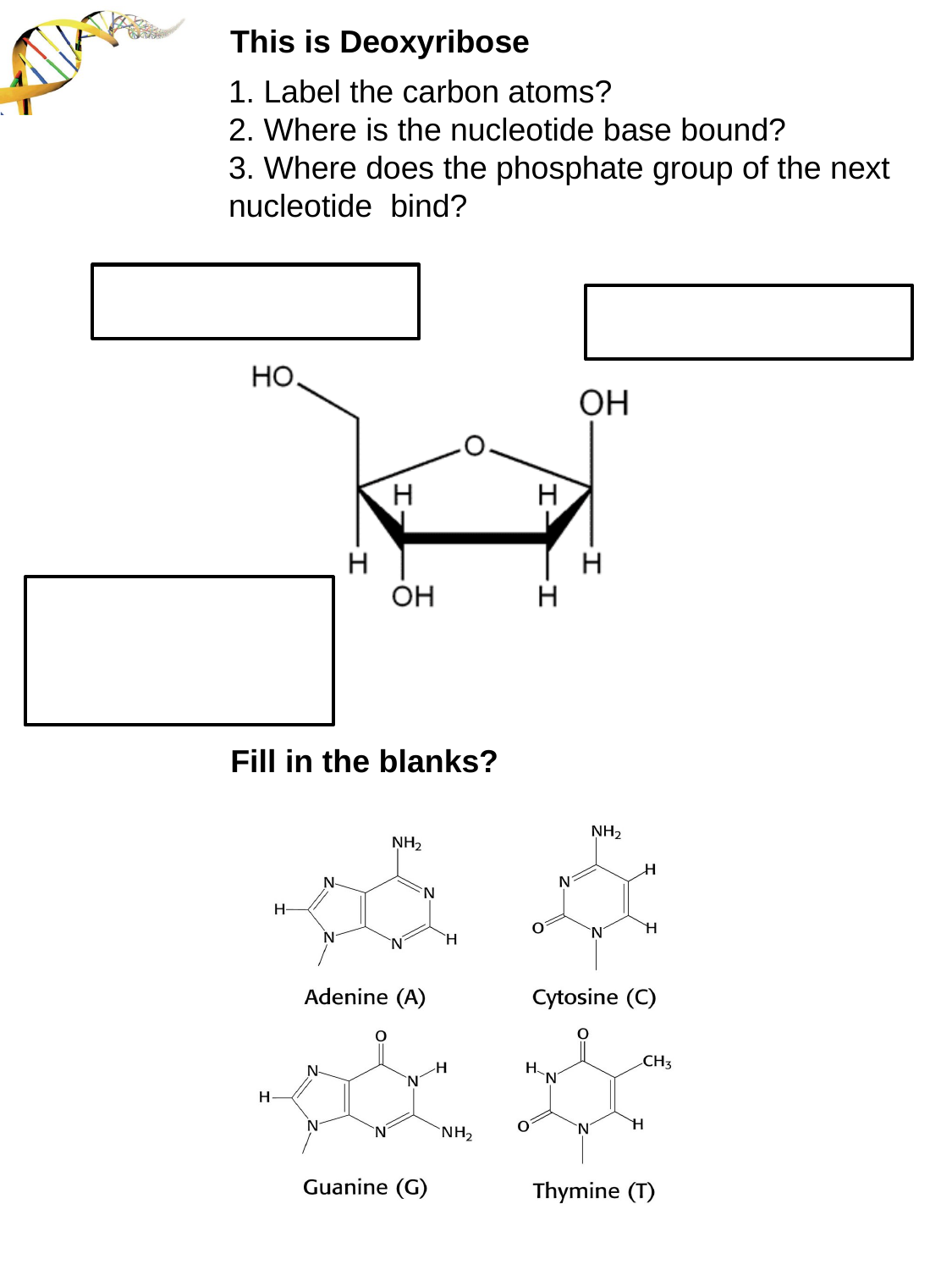

This is Deoxyribose
1. Label the carbon atoms?
2. Where is the nucleotide base bound?
3. Where does the phosphate group of the next nucleotide bind?
Fill in the blanks?

## Slide 4
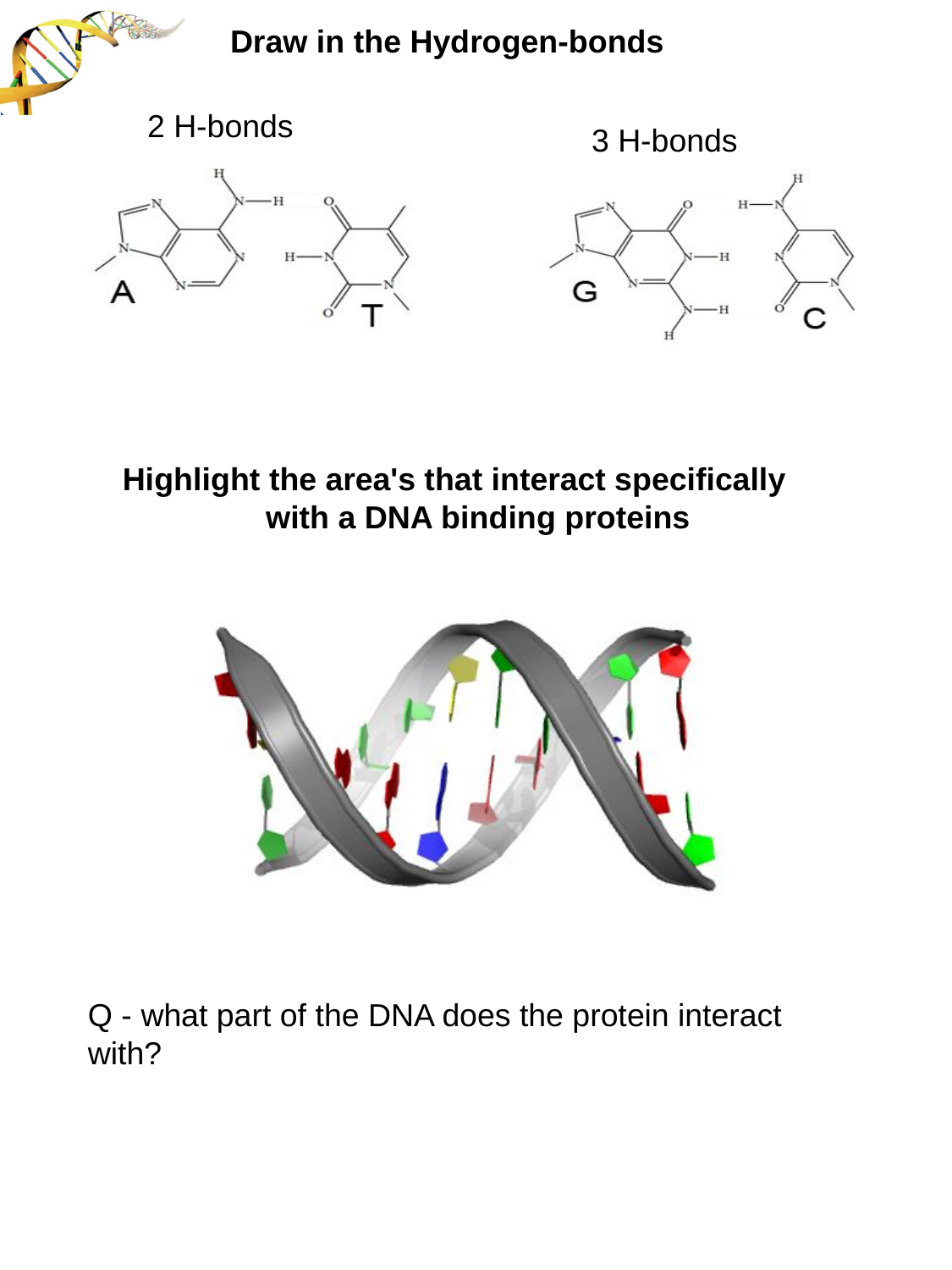

Draw in the Hydrogen-bonds
2 H-bonds
3 H-bonds
Highlight the area's that interact specifically with a DNA binding proteins
Q - what part of the DNA does the protein interact with?
