# Supplementary material for: Active learning in the lecture theatre using 3D printed objects
Source: F1000Res. 2016 Jun 3;5:61. Originally published 2016 Jan 13. [Version 2] doi: 10.12688/f1000research.7632.2 (PMC4911624; doi:10.12688/f1000research.7632.2)
Supplement: Supplementary file 6 [file f1000research-5-9453-s0005.tgz › d80ddc64-cc47-4cb9-b6d4-a1d7b8b3de3f.docx]

**Do you remember using the 3D printed models?**

**Yes - 35**

**No - 9**

**Did you find the models helpful if so how?**

**Positive Comments.**

Yes they were helpful as a demonstration of the structure of DNA and its configuration. Also they were a good practical method to remember the major and minor groves

Yes, provided a more practical approach, made it easier to understand and picture the DNA and grooves

Yes can visualise the special arrangement of molecules much easier. Much easier to see how binding occurs such as enzyme models

Yes because it's hard to visualise abstract concepts

easy to visualise structures and how they fit together, which is otherwise difficult from just a picture on a screen

Helped to actually visualise what the lecture was about, made understanding much easier

Allowed to properly visualize major and minor grooves of DNA

very different to normal lectures, sticks out in the mind because of it

visualising 3D protein structures in the flesh makes understanding easier

yes because you are able to see the dimensions and have a close enough actual presentation of the molecules before you

they were very useful for highlighting the key lecture points as well as being a visual aid. Still remember their use in the lecture 3 years later

visually showed groves in DNA strands - help people who learn using visual prompts. Engaging explains well what is needed from it

very helpful to actually hold the model to understand the structure. Still remember using these three years later so obviously helped me to remember

visualise the structure, easier to learn by actually doing it

I found them useful in being able to visualise them

fun and cool way to learn

it was helpful to understand the difference between the major and minor grooves in DNA

understanding DNA shape and orientation, very useful to help understand major and minor grooves

these amazing and made proteins fun

allowed us to visualise DNA as a 3D structure and understand it more. eg major and minor grooves. Visualising molecules / structures on 2D screen isn't always easy to understand

helped me visualise structure and active site during enzymology

allowed us to visualise the major and minor groove of DNA, as well as the binding sites for enzyme or ribosome

allowed me to visualise the structures and hold them in my hands. Better than 2D picture / diagrams in a text book or on the internet

helped to visualise the structures, nice addition to a lecture, helped to enhance memory. 3D models are much better than simple 2D drawings

they gave a 3D better understanding of the 3D structure of the enzyme than a 2D computer image

because it helped me understand how chemical structure correlated with 3D structure

allow to visualise the 3D shape, instead of a 2D image

easier to visualise shape how things fir together

hands on good for visual learners

helped understand structure better

easier to visualise

yes they gave me a helpful visual aid for exam revision

**Neutral Comments**

ish, useful to visualise active sites and substrate binding but has limited usefulness. Handy visual aids

kind of, it made the lecture a little different and hence memorable

passed around

**Should the 3D models be used more Y/N?**

**Y - 35**

**N - 0**

Yes. Is a useful model for learning

can use with other proteins to find active sites ect

incorporate colours or something to obtain more information out of it

any visual aid is helpful

because it's cool, but also it brings the molecule to life - something different (interactive learning)

models can help simplify complicated concepts

they were really useful to help visualise details for the exams

when appropriate of course

could help students understand scale eg Golgi apparatus bigger than a ribosome, wakes students up and makes lectures more interesting

include other examples eg organelles like Golgi
