# Supplementary material for: Active learning in the lecture theatre using 3D printed objects
Source: F1000Res. 2016 Jun 3;5:61. Originally published 2016 Jan 13. [Version 2] doi: 10.12688/f1000research.7632.2 (PMC4911624; doi:10.12688/f1000research.7632.2)
Supplement: Peer review history

# Peer review: Referee response for version 2

**DOI:** 10.5256/f1000research.9453.r14156

**Published:** 2016-06-15

**Authors:** Carola Bruna

**Affiliations:** 1 Faculty of Biological Sciences, University of Concepción, Concepción, Chile

The author has provided an improved version of the manuscript in which he has considered most of the suggestions and commentaries from the referees. He has improved the discussion regarding the advantages of the use of 3D printed objects in comparison with other alternatives, has described the actual activity further and attempted to assess the perception of the students.

 Experiences in the classroom in which no learning gain is demonstrated or there is no new theoretical proposal are difficult to publish, although some of these experiences can be very useful to design and implement new learning environments. In this case, the use of 3D printed options in a large classroom is presented, a technology that will certainly be a part of the near future of education to help students visualize and understand complex concepts. That being said, there are still some issues to be addressed regarding the actual implementation, that I consider the author will be easily able to clarify:

- This was implemented in the first year of study with cohorts of 150 students for three consecutive years, while the assessment was done in the final year of study of 44 students. Why only 44? Is it a 3 year study program and only the first cohort was surveyed?
- The paragraph in which Kolb’s experiential learning cycle is presented should be discussed further or eliminated. As it is, it provides insufficient information, confusing the reader.
- In the Doing section it states “apply new knowledge…”, what new knowledge? The experiential approaches followed a lecture in which the concepts were previously presented? It should be clarified if the models were used before, during or after presenting the theory and concepts involved.

I consider this manuscript approved. Nevertheless, that these minor issues should be addressed in order for this experience to be a valuable approach for other educators.

I have read this submission. I believe that I have an appropriate level of expertise to confirm that it is of an acceptable scientific standard.

# Peer review: Referee response for version 2

**DOI:** 10.5256/f1000research.9453.r14155

**Published:** 2016-06-13

**Authors:** Richard Bowater

**Affiliations:** 1 School of Biological Sciences, University of East Anglia, Norwich, UK

This article is a revised version of an earlier submission. In submitting this revised version, the author has addressed the major concerns from two reviewers. The comments have been addressed in a positive and constructive way, and the new version is significantly improved. The author has provided careful responses to the comments from both reviewers, which highlights the major changes that have been introduced in the revised submission.

 Particular improvements in the manuscript include:

- a more careful description of the arrangements for the sessions including their costs;
- some qualitative evaluation of the approach by the students involved in the sessions;
- a deeper introduction to the literature that describes this type of approach, including in other disciplines.

Significantly, the title of the article has been changed and it has now been submitted as a Research Note, rather than its original submission as an Observational Article. These changes seem appropriate.

 One minor comment is that the responses to the two reviewers seem to have been swapped between the reviewers i.e. the response to Reviewer #1 is actually the response to comments by Reviewer #2 and vice versa. Since both sets of comments have been addressed, ultimately this does not cause problems in working out why changes have been made.

I have read this submission. I believe that I have an appropriate level of expertise to confirm that it is of an acceptable scientific standard.

# Peer review: Referee response for version 1

**DOI:** 10.5256/f1000research.8219.r12885

**Published:** 2016-03-14

**Authors:** Carola Bruna

**Affiliations:** 1 Faculty of Biological Sciences, University of Concepción, Concepción, Chile

This article presents a pedagogical experience in which 3D printed objects were used to promote the understanding of biochemistry concepts in two student-centred interactive sessions within a course. The design is based in the advances and availability of 3D printing technology and the importance of conceptualizing three-dimensional structure in order to understand biochemistry core concepts such as the relationship between the structure and function of proteins.

3D printing technology is in fact becoming increasingly accessible and it is contributing and changing many fields. Undoubtedly, education should benefit from this technology, probably many teachers are thinking about it, including myself. Thus, experiences on how to implement it in class are interesting and valuable. In this sense, this work can contribute with relevant information. Nevertheless, some aspects need to be addressed, clarified or discussed further so that weaknesses and strengths are identified, which will allow replicating or improving the shared experience. In this context, the following suggestions could improve this manuscript:

 

- Title: The work is more than using 3D printed objects, it is student-centred education, interactive and motivating and specially thought to address a problem in science, to enable visualization of abstract concepts, specifically in Biochemistry.
- The author state that 3D structures and related problems have been presented with slides. Many teachers also use 3D visualization programs, combining theoretical and computer laboratory sessions. Differences, advantages and disadvantages between these two approaches could be discussed.
- The experience should be described further. What was actually done? Why were those structures chosen? Which were the concepts students were expected to learn in each session? What were the related learning outcomes? How many groups? How many molecules? Was the discussion tutored? If it was, how many tutors participated? The models seem very small, what were the structural features they had to identify mentioned in figure 3? Costs?
- As this experience was justified as a mean for conceptualizing 3D shapes for understanding Biochemistry, other reported experiences using molecules should be discussed and compared. For example: “The effect of student manipulation of molecular models on chemistry achievement according to Piagetian level” or “Using three-dimensional models to teach molecular structures in high school chemistry”. I am sure there are more examples, maybe even in biochemistry.
- The main weakness of this work is that no assessment of the approach is included. I understand that it is very difficult to assess learning gain, and that the intention of the author is modest, in the sense that this article is intended to share an experience instead of researching education. However, at least the perception of the students should have been included. This would at least suggest the strongest aspects and allowed identifying possible improvements. If the intervention is recent and there still contact with the students, there is still a possibility to conduct an online survey. If that is not the case, at least the author should comment further regarding the perception of the teacher: Did the students seem motivated? Did they participate more actively than in other activities? Did they comment informally positive aspects? Another approach could be to analyze exams answers regarding the concepts that were studied in these special sessions and compared them to those of the previous year.

 

Despite the previous comments, I still consider that sharing this experience is valuable to the community. In this work, I particularly appreciate that the activity was implemented in a large course using a standard classroom, with no additional requirements. If the main suggestions could be addressed, I believe it would be appropriate for indexing as published in *F1000Research.*

I have read this submission. I believe that I have an appropriate level of expertise to confirm that it is of an acceptable scientific standard, however I have significant reservations, as outlined above.

# Response

**Published:** 2016-05-12

**Authors:** David Smith

**Affiliations:** Sheffield Hallam University, UK

Thank you for your comments and by addressing them I hope the manuscript has been improved. In the response below I have set out the changes made. Some off the comments made are reflected by referee 2 and the response below is applicable to both referees.

**Comment - However, there is no evaluation of whether the approach is successful or cost-effective and, thus, readers cannot easily judge if they should consider it in their own teaching environment**.

The cost per model was around £7 when purchased internally which covered materials. The models have now been used over four years in two different streams (Biology and Chemistry). There 20 models were produced with a total cost of £140. This is less than the printing cost of the handouts for the same session.

**Comment - There is a comment that the author “observed that this approach led to increased student engagement in the lecture theatre as the students are willing to talk with each other and the lecturer as confidence in their understanding increased”, but there is no direct feedback from students involved in the sessions.**

On the advice of the reviewer the perception of the students was assessed via a written questionnaire. Students in their final year of study were asked to reflect on the use of the models during their past learning. 44 students were questioned and 35 remembered using the models. 32 of these responded with positive comments and 3 with neutral comments. Of the 9 students who did not remember using the models many admitted to not having attended the lecture.  The following text and supplementary information has now been added. In addition comments have been referred to in the discussion section.

Results

Students in their final year of study were asked to reflect on the use of the 3D printed models during their past learning. 44 students were asked "Did you find the models helpful, if so how?" through an anonymous survey. Of the 44 responses 35 remembered using the models and 32 of these responded with positive comments (3 with neutral comments). Of the 9 students who did not remember using the models many admitted to not having attended the lecture.

Some students commented on the benefits the models provided as a visual aid,

*they were very useful for highlighting the key lecture points as well as being a visual aid*."

Student comments also highlighted the use of the models as a tool in understanding the key learning objectives,

" *Very useful to help understand major and minor grooves*", and

" *Allowed us to visualise the major and minor groove of DNA, as well as the binding sites for enzyme*".

The students also explained that the models provided an alternative way of presenting information,

*"they gave a 3D better understanding of the 3D structure of the enzyme than a 2D computer image.*"

It was also noted that the models could be used to gain a sense of scale, for example, the difference in size between a ribosome and organelles. The full list of comments is included as supplementary information. During each of the sessions there was a high level of student engagement involving the students talking to each other and to the lecturer with the key learning points being recalled three years after the teaching session.

**Comment -The article suggests that its approach would be of value to other disciplines. In fact, the use of artefacts and the potential to handle objects is frequently used within the arts and humanities, particularly in subjects that combine history and art. The benefits of such approaches have been well described in the pedagogical literature for these disciplines and this article would be improved if it provided a deeper review and analysis of such information.**

The following text has been integrated into the introduction discussing object based learning theory.

The handling of molecular models within teaching sessions can aid this mental rotation and the use of models falls within the theories of object based learning. This approach involves the active integration of objects into the learning environment (Chatterjee, 2015). Working with objects strengthens learning is the central proposition of object-based learning as, according to Romanek (2008), the sense of touch can lead to a more memorable learning experience. The use of museums artefacts in history, art and biology has been well explored and there are a number of parallels between the handling of objects within these disciplines. Object- based learning theory links student activity to meaning making by challenging the student to  engage with and interrogate the object It represents a constructivist approach in which the students develop their knowledge and understanding though interaction (Chatterjee, 2015). While the teacher facilitates this learning, the students have to learn for themselves through their interaction with each other centred on the object (Hannan, 2013). This approach enables the student to explore processes and events related to the object and further link these observations to complex abstract ideas and concepts.

Findings from my study support Chatterjee's (2015) conclusion that the students gain real knowledge by being actively involved in the experience of handling the objects. It has been highlighted that object-based learning should be both mentally and physically stimulating through some form of problem solving or experimentation (Coffield, 2004) and this was achieved in this study by challenging the students to find the active site of Lysozyme or identify the region of DNA to which proteins bind.

While objects can be used to enhance learning, there are logistical and pedagogical barriers to implementing them. For example, Cain (2010) says the main barrier for the implementation in the biosciences within the lecture theatre is the change to the adoption of a more student-centred and open-ended activities. The use of models in teaching needs to expand the concepts as understood by students at a particular stage in their learning (Bent, 1984) There is a danger for example when discussing enzymes that concepts such as the lock and key model could be reinforced over an induced fit model. Such dangers should be at the forefront of the lectures mind when utilising these models.

# Peer review: Referee response for version 1

**DOI:** 10.5256/f1000research.8219.r11937

**Published:** 2016-01-26

**Authors:** Richard Bowater

**Affiliations:** 1 School of Biological Sciences, University of East Anglia, Norwich, UK

This article provides a brief description of an attempt to introduce experiential learning into lectures in the biochemical sciences. As described below, the author highlights a fundamental aspect of the subject that some students struggle to understand fully and proposes an approach that aims to help students engage better with the topic and the issues involved.

Structures of biological molecules are critical to their function and this is a core principle that must be understood by all students of the field of biochemistry. Full appreciation of this link requires students to develop 3-dimensional knowledge of a molecule and relate this to its biochemical activities. These are challenging concepts to introduce to students, especially in large cohorts. This article describes a strategy that takes advantage of the increasing availability and cost effectiveness of 3D printers to prepare molecules that can be “handled” by the students. The 3D printed biological molecules are used as active learning tools to stimulate student engagement and a deeper understanding of the link between structure and biochemical function. The approach described in the article is that core knowledge should first be established through conventional teaching approaches. The teaching delivery is then supplemented by discussions among the students and lecturer about the printed molecules, allowing interrogation of its physical environment.

The article provides an appropriate discussion of a topic that is clearly challenging to inexperienced learners in the biochemical sciences and suggests an approach that goes beyond the conventional lecture. However, there is no evaluation of whether the approach is successful or cost-effective and, thus, readers cannot easily judge if they should consider it in their own teaching environment. There is a comment that the author “observed that this approach led to increased student engagement in the lecture theatre as the students are willing to talk with each other and the lecturer as confidence in their understanding increased”, but there is no direct feedback from students involved in the sessions.

The article suggests that its approach would be of value to other disciplines. In fact, the use of artefacts and the potential to handle objects is frequently used within the arts and humanities, particularly in subjects that combine history and art. The benefits of such approaches have been well described in the pedagogical literature for these disciplines and this article would be improved if it provided a deeper review and analysis of such information.

To be of improved value to the biochemistry teaching community, this study needs to provide a much clearer hypothesis. What is (are) its fundamental aim(s) and how will its success be tested and evaluated? It would be beneficial if a study could be developed that tested whether inclusion of the printed molecules in teaching sessions led to improved biochemical understanding among the students involved. Clearly this could take a significant amount of time and effort and may be beyond the remit of the study that is described.

Whether or not it is possible to evaluate the success of the approach, there are a number of important details that would be useful to assist other educators in considering whether they should adopt the strategy. For example, how many molecules are needed for each group of student and what is the approximate cost of preparing the molecules? Is there any value in using the approach multiple times (with different molecules) within the same module or same cohort of students?

In relation to publication of this article, it is not clear that it has been submitted as the most appropriate type of article for *F1000Research*. The study has been submitted as an Observation Article and the guidelines to authors state that: “Observation Articles allow the description of a novel observation that may be unexpected, and possibly currently without explanation.” It is not clear to me what details in the article are “unexpected” or “without explanation” - these points need to be made much clearer to the reader. In considering the different types of articles published within *F1000Research*, it seems that it may be more applicable as a Research Note.

The article is reasonably clear and well written, but there are some minor errors that should be corrected by careful proof-reading. The title could be improved by highlighting that it focuses on “biochemistry teaching”.

In summary, this article describes an interesting but rather straight forward intervention to allow biochemistry students to gain a better appreciation of the link between molecular structure and function. The suggested approach makes good use of computer-based technologies that are becoming more accessible and it is thus likely to be of interest to a wide group of educators in the biosciences (and other disciplines). Unfortunately, the study does not present any evaluation of the effect of the approach on student learning or understanding. Such information should be obtained from further cohorts of students in order to enable other educators to consider the value in the approach.

I have read this submission. I believe that I have an appropriate level of expertise to confirm that it is of an acceptable scientific standard, however I have significant reservations, as outlined above.

# Response

**Published:** 2016-05-12

**Authors:** David Smith

**Affiliations:** Sheffield Hallam University, UK

Thank you for your comments and by addressing them I hope the manuscript has been improved. In the response below I have set out the changes made.

**The work is more than using 3D printed objects, it is student-centred education, interactive and motivating and specially thought to address a problem in science, to enable visualization of abstract concepts, specifically in Biochemistry.**

Thank you for your suggestions and I have altered the title to reflect your comments. "Active learning in the lecture theatre using 3D printed objects to visualise abstract concepts."

**Many teachers also use 3D visualization programs, combining theoretical and computer laboratory sessions. Differences, advantages and disadvantages between these two approaches could be discussed.**

The use of computer based representations is now easily accessible via personal devices. There are a wide range of 3D visualisation programs available which enable molecular structures to be moved, altered, rotated and interrogated. Programs such as the Java based Jmol applet allow students to manipulate molecules and investigate their structures. There are also a range of free programs that allow students to visualise molecules on smart phones and tablets such as "Molecules" for Apple iOS and "ESmol" for Android devices. These programs offer many advantages such as ready access to the > 118500 structures available in the protein data bank (PDB). However, their use as tool in large group teaching can be problematic as all students require access to a device. In addition providing technical support to 150 students across three different platforms within a lecture theatre can also be problematic. Molecular viewers do work very well in small group seminar or lab sessions where support can be given or devices can be provided. Students can be directed through a range of tasks and the molecules can be explored in greater depth (Harris, 2009). However, the tablet based applications lack the tactile aspect of physically handling the object in question and can prevent the abstract observations that generate the initial interactions. The models are also engaging and can be used by groups of people who have had little training, unlike the visualisation programs (Harris, 2009). The models also allow an ability to quickly make abstract observations for example the spiral nature of DNA or the large clefts on an enzyme. It is these abstract observations that  stimulate the initial peer-to-peer conversations. This approach allows the students to remain engaged with the topic and revisit their observations after the teaching session.  In a study in which 3D printed models were offered alongside a molecular imaging program the students tend to prefer the models when asked questions about molecular structure that required higher order thinking skills (Harris, 2008). The tactile nature of the models appears therefore to leads to a more lasting memory of the interaction (Hurman, 2006).

**The response to the following questions has been embedded directly into the text.**

**What was actually done?**

Molecules were handed around the group by the lecturer. They were handed out starting at the end of each of the rows of students. The students were asked to pass the molecules around and handle them directly. Once most of the students had handled the objects they were encouraged to talk with their peers about their observations.

**Why were those structures chosen?**

The two structures were chosen as they these molecules are established as examples within the taught material on the course. DNA is central to molecular biology and an understanding of how other molecules such as transcription factors interact with it is a key learning point in the Biochemistry program. Lysozyme was chosen as its structure and function are well understood. It also has a defined active site in which the substrate has been modelled. The use of lysozyme also integrated well with the rest of the module. Its action on the lipid polysaccharides found on the cell wall of gram negative bacteria resulting in lysis allowed links to microbiology and protein purification. It also integrates well with a second year structural biology practical in which lysozyme can be induced to undergo change from an alpha / beta structure to the beta sheet rich structure of amyloid fibrils resulting in a loss of function.

**Which were the concepts students were expected to learn in each session?**

The main concepts that the students were expected to learn was that the structure of a molecule brings about its function and subsequent properties.

**What were the related learning outcomes?**

In the DNA sessions the main learning outcome was to understand the difference between the major and minor grove within the structure of DNA and how proteins interact with these groves. Within the lysozyme sessions the learning outcome was to be able to identify where an active site maybe located on a protein and how a substrate would interact with it.

**How many groups?**

The session has been run twice a  year for three consecutive years with ~120 - 150 students being present at each session.

**How many molecules?**

Within each session 20 molecules were used.

**Was the discussion tutored? If it was, how many tutors participated?**

The sessions were run in a standard lecture theatre with one lecturer delivering the session.

**The models seem very small, what were the structural features they had to identify mentioned in figure 3?**

For the DNA molecule it was the major and minor grove. The models are scaled such that an index finger would fit into the major grove. In the case if the enzyme it was the active site located across the middle of the protein.

**Costs?**

£7 each for raw materials

**As this experience was justified as a mean for conceptualizing 3D shapes for understanding Biochemistry, other reported experiences using molecules should be discussed and compared.**

The following text has been added

The use of physical models as a learning tool have been used to depict molecular geometry in both biochemistry and chemistry (Dori, 2001). The benefit of these objects has been demonstrated in an analysis of visuospatial thinking in chemistry. In this study it was concluded that adept visual perception skills correlate with achievement. DNA and protein models assembled from coloured computer-printouts on transparency film sheets have also been demonstrated to aid students in grasping various aspects of biopolymers (Jittivadhna, 2010). Students who hold physical models in their hands gain a better understanding of molecular geometry, than they could achieve solely from viewing images on a printed page. It has been reported by both Herman (2006) and Bain (2006) that physical models that can be easily manipulated, can play an important role in capturing the interest of students and encouraging deeper sophisticated thinking (Bain, 2006). Additionally, the students gain a language for talking about the concepts in question and enhance their understanding of abstract concepts by handling models (Wu, 2004).

The positive comments from students polled in this study, in which 3D printed models were rated as most useful, are echoed in a comparable study where a side-by-side comparisons of seven different learning tools were undertaken by students in an introductory biochemistry class (Roberts, 2005). In that study, the models were shown to have an important role in capturing the interest of the students and stimulated sophisticated questioning. The main difference between these observations and those presented here, relate to the learning environment. Within the side-by-side comparisons study, the core concepts were introduced in a lecture and the models were handled as part of a 3-wk laboratory in a group of twenty students, generating tangible learning gains were observed. In the study discussed here, however, 150 students were allowed to handle the models directly within the lecture environment. Handling the models aided the students in visualising the molecules demonstrating the applicability of this approach to large group teaching. Schönborn, (2006) states that the ability to visualise ideas is a key skill for all students and it is a key skill for biochemists who are often presented with a range of visual interpretation including drawings, images, dynamic visuals, animated visuals, multimedia, and virtual reality environments. The use of models has the potential to help students construct their own visualisation and understanding of these molecules as demonstrated by the student comments reported here.

**The main weakness of this work is that no assessment of the approach is included.**

On the advice of both reviewers the perception of the students was assessed via a written questionnaire. Students in their final year of study were asked to reflect on the use of the models during their past learning. 44 students were questioned and 35 remembered using the models. 32 of these responded with positive comments and 3 with neutral comments. Of the 9 students who did not remember using the models many admitted to not having attended the lecture.

The following text and supplementary information has now been added. In addition comments have been referred to in the discussion section.

Results

Students in their final year of study were asked to reflect on the use of the 3D printed models during their past learning. 44 students were asked "Did you find the models helpful, if so how?" through an anonymous survey. Of the 44 responses 35 remembered using the models and 32 of these responded with positive comments (3 with neutral comments). Of the 9 students who did not remember using the models many admitted to not having attended the lecture.

Some students commented on the benefits the models provided as a visual aid,

"they were very useful for highlighting the key lecture points as well as being a visual aid."

Student comments also highlighted the use of the models as a tool in understanding the key learning objectives,

"Very useful to help understand major and minor grooves", and

 "Allowed us to visualise the major and minor groove of DNA, as well as the binding sites for enzyme".

 The students also explained that the models provided an alternative way of presenting information,

"they gave a 3D better understanding of the 3D structure of the enzyme than a 2D computer image."

It was also noted that the models could be used to gain a sense of scale, for example,s the difference in size between a ribosome and organelles. The full list of comments is included as supplementary information. During each of the sessions there was a high level of student engagement involving the students talking to each other and to the lecturer with the key learning points being recalled three years after the teaching session.

**Did the students seem motivated?**

Yes there was a high level of engagement during the sessions; the students were talking to each other and to the lecturer.

**Did they participate more actively than in other activities?**

The participation level was probably equivalent to other sessions. However I have noted that a range of activities within the sessions a good way to keep students motivated and engaged. Too much of any intervention can lead to disengagement as has been reported for flipped classroom approaches.

**Did they comment informally positive aspects?**

See above response

**Another approach could be to analyze exams answers regarding the concepts that were studied in these special sessions and compared them to those of the previous year.**

Unfortunately we do not hold the exam scripts locally and so gaining access to this is not possible. However it will be built into further studies as it is a good suggestion.
